# Habitat provided by native species facilitates higher abundances of an invader in its introduced compared to native range

**DOI:** 10.1038/s41598-020-63429-2

**Published:** 2020-04-14

**Authors:** Paul E. Gribben, Alistair G. B. Poore, Mads S. Thomsen, Phoebe Quesey, Emma Weschke, Jeffrey T. Wright

**Affiliations:** 1 Centre for Marine Science and Innovation, School of Biological, Earth and Environmental Science, University of New South Wales, Sydney, NSW 2052 Australia; 2 Sydney Institute of Marine Science, 19 Chowder Bay Road, Mosman, NSW 2088 Australia; 3 Evolution and Ecology Research Centre, School of Biological, Earth and Environmental Science, University of New South Wales, Sydney, NSW 2052 Australia; 4 Marine Ecology Research Group and Centre of Integrative Ecology, School of Biological Sciences, University of Canterbury, Christchurch, New Zealand; 5 Institute for Marine and Antarctic Studies, University of Tasmania, Private Bag 129, Hobart, 7001 Australia

**Keywords:** Biogeography, Invasive species, Biogeography, Invasive species

## Abstract

The impacts invasive species have on biodiversity and ecosystem function globally have been linked to the higher abundances they often obtain in their introduced compared to native ranges. Higher abundances of invaders in the introduced range are often explained by a reduction in negative species interactions in that range, although results are equivocal. The role of positive interactions in explaining differences in  the abundance of invaders between native and invasive ranges has not been tested. Using biogeographic surveys, we showed that the rocky shore porcelain crab, *Petrolisthes elongatus*, was ~4 times more abundant in its introduced (Tasmania, Australia) compared to its native (New Zealand) range. The habitat of these crabs in the invaded range (underside of intertidal boulders) was extensively covered with the habitat-forming tubeworm *Galeolaria caespitosa*. We tested whether the habitat provided by the tubeworm facilitates a higher abundance of the invasive crab by creating mimics of boulders with and without the tubeworm physical structure and measured crab colonisation into these habitats at three sites in both Tasmania and New Zealand. Adding the tubeworm structure increased crab abundance by an average of 85% across all sites in both ranges. Our intercontinental biogeographic survey and experiment demonstrate that native species can facilitate invader abundance and that positive interactions can be important drivers of invasion success.

## Introduction

Historically, the processes structuring ecological communities have been viewed through the lens of negative biotic interactions. Understanding the ecology of species invasions has developed along parallel lines. For example, the impacts of invasive species on native biota via competition and predation are well documented^1,2^ and changes in these negative interactions are also important drivers of invader abundances at biogeographic scales^3^. The loss of natural enemies (e.g. parasites, pathogens and predators^4–6^); and the evolution of increased competitive ability^7–9^ can explain higher abundances of invaders in their introduced compared to the native range. The past 20 years, however, have seen positive interactions as an emerging paradigm for structuring ecological communities^10–14^ and, in fact, positive interactions have as strong effects on community structure as better studied negative interactions^15^. In relatively few instances, positive interactions between native and invasive species^16–18^ and co-occurring invasive species^19,20^ can determine invader abundance in their introduced range but none of these studies have tested if and how facilitation may also affect the invasive species in its native range. Whether changes in positive interactions at biogeographic scales increase invader abundances in their introduced compared to native range has not been tested.

Whilst the enemies left behind in native ranges can lead to increases in abundance of invaders in introduced ranges, the native species in the introduce range can also increase invader abundances. For example, invasive plants can acquire pollinators which ensure reproductive success^21^ and soil mu tualists such as mycorrhizal fungi and nitrogen‐fixers, which improve the nutrient status of their host-plants, lead to higher abundances of plants in their introduced compared to native range^22^. In addition, the provision of habitat by ecosystem engineers (sensu^23,24^) can also facilitate invader abundances. Native terrestrial plants can facilitate invasive plants^25^ while native marine mussels, seaweed and saltmarsh facilitate invasive seaweeds and mobile invertebrates^26–29^. To date, these studies have focussed on positive interactions in the new range only, and whether changes in positive interactions – either in their frequency or intensity – between native and introduced ranges also explains higher abundances of invaders in their introduced range remains unexplored.

To address this knowledge gap, we conducted biogeographic-scale surveys and an experiment to determine the  mechanisms underpinning the abundance of the invasive porcelain crab *Petrolisthes elongatus* in both its native (New Zealand) and introduced (Tasmania, Australia) ranges. Following its introduction into Tasmania in the early 1900s via ballast rock or the live oyster trade between the two countries^30,31^, *P. elongatus* spread rapidly and is now widespread and a dominant member of intertidal rocky shore communities^32,33^. Biogeographic surveys show that *P. elongatus* is more abundant in its invaded compared to native range^34^. Furthermore, studies from its invaded range only show the abundance of adults and new recruits of *P. elongatus* are positively correlated to the amount of boulder habitat, and are further facilitated by the presence of the native calcareous tube-forming serpulid worm, *Galeolaria caespitosa*^28,32,35,36^. *G. caespitosa* forms a complex habitat on the underside of boulders^28^ which reduces temperature stress beneath boulders^33^. Thus, higher abundances of *P. elongatus* in Tasmania compared to New Zealand may be due to the differences in the amount of boulder habitat for colonisation and/or the additional habitat provided by this native ecosystem engineer between the introduced and native ranges.

**Figure 1:**
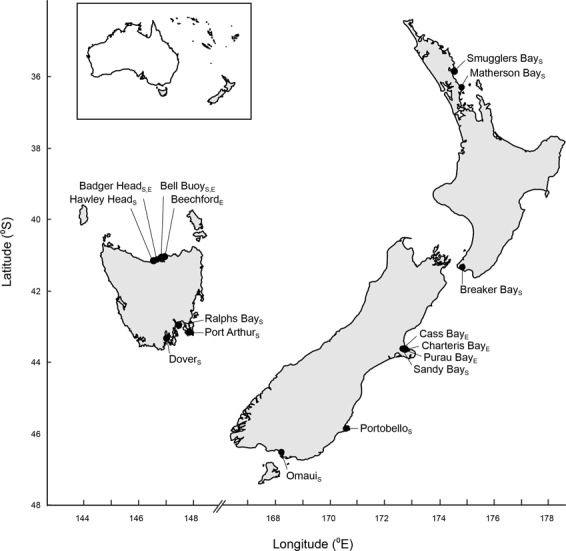
Map of the 12 survey and six experimental sites in the introduced (Tasmania) and native (New Zealand) range of *Petrolisthes elongatus*. Site names with the subscript S were used in the survey and those with the subscript E were used in the experiment that manipulated the tubeworm structure.

We conducted structured surveys to quantify *P. elongatus* abundance and habitat characteristics throughout Tasmania and New Zealand (Fig. 1). We then conducted a standardised *in situ* experiment at multiple sites to experimentally determine the facilitative role of tubeworm structuresin both ranges using mimics of boulders with and without the habitat structure created by *G. caespitosa* on their underside. Whilst surveys and meta-analyses provide evidence consistent with natural enemies having lower impacts in the introduced region^5^, experiments conducted across both ranges to confirm these patterns are rare despite being critical for elucidating the underlying mechanisms^37–41^. Currently, no studies have manipulated the presence of species that may facilitate invaders in both the native and introduced ranges to determine whether positive interactions differ between regions. We provide the first test of the prediction that positive effects are more frequent in the invasive compared to native range.

## Results/Discussion

**Figure 2:**
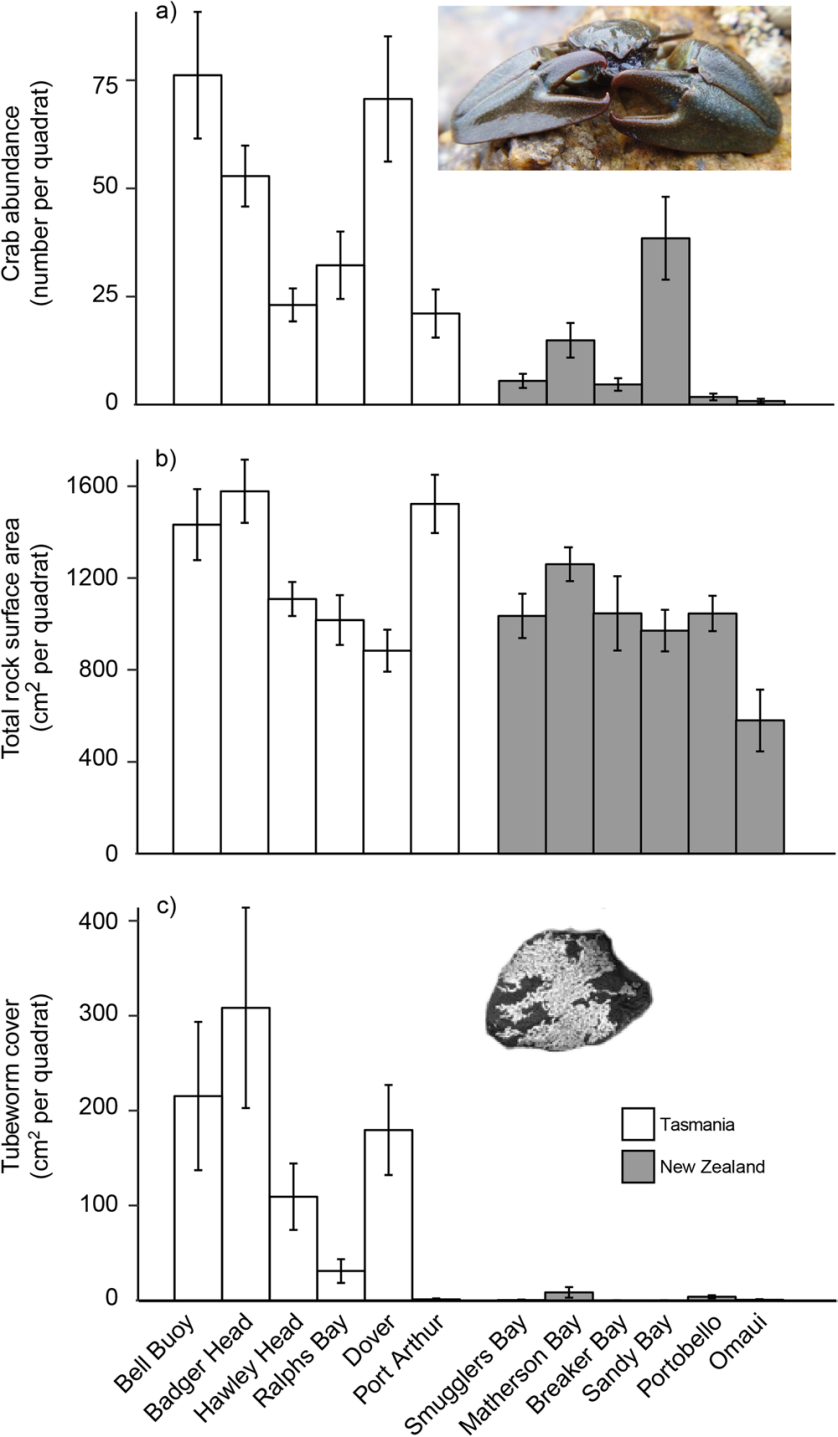
The abundance of *Petrolisthes elongatus* (a), total underside surface area of boulders (b) and total underside cover of *Galeolaria caespitosa* (tubeworm cover; c) per quadrat at six sites in the introduced (Tasmania) and native (New Zealand) range of *P. elongatus*. Data are means ± SE (n = 15–18 quadrats per site except Omaui, n = 6).

Our biogeographic surveys showed the abundance of *P. elongatus* was higher in its introduced range compared to its native range (Fig. 2a), supporting the findings of an earlier biogeographic survey^34^. Our survey contained seven sites (five in Tasmania and two in New Zealand) not previously surveyed in^34^ indicating that higher abundances of *P. elongatus* in Tasmania are temporally and spatially consistent. This study extends previous research to show that increased abundances in the introduced range are related to higher cover of the habitat-forming ecosystem engineer *G. caespitosa* in Tasmania. Tubeworm cover in New Zealand, which consisted of *G. caespitosa* and/or the morphologically similar *Spirobranchus cariniferus* (we did not distinguish between the two species because they have very similar tube structures), was extremely low. Our experiment confirmed the positive effect of *G. caespitosa* on *P. elongatus* local abundance, thus providing the first experimental evidence that more frequent positive interactions, in our case due to the increased habitat-complexity provided by a facilitating species, determines the higher abundance of an invasive species in its introduced vs. native range.

**Figure 3:**
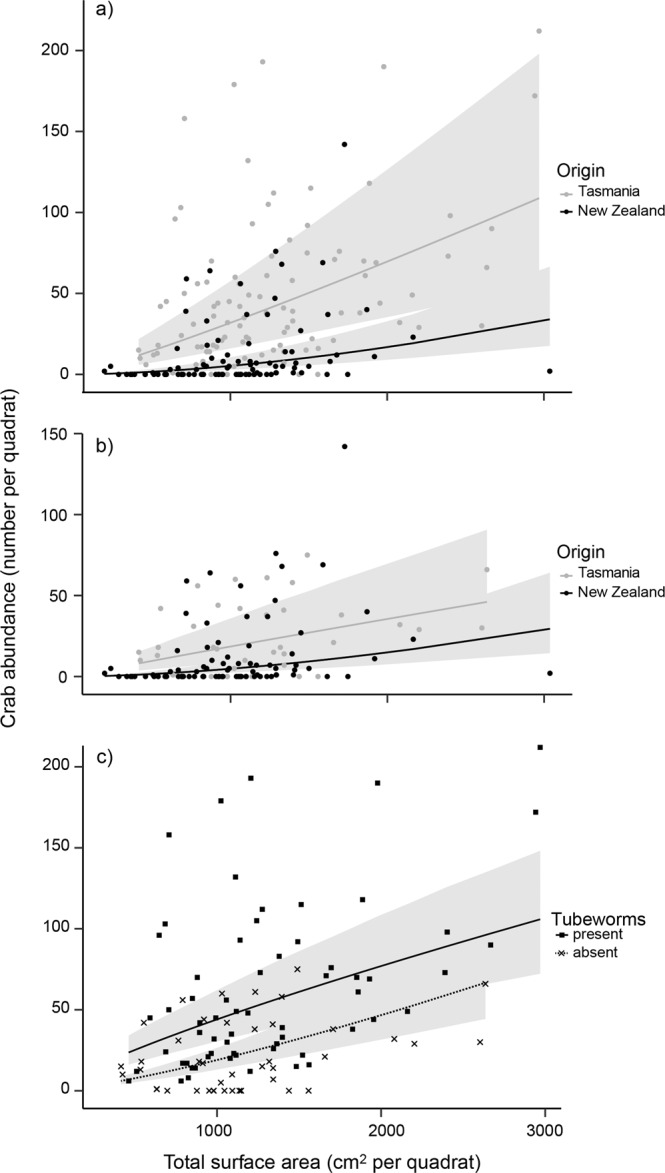
The relationships between abundance of *Petrolisthes elongatus* and total underside surface area of boulders per quadrat for all quadrats in the introduced (Tasmania) and native (New Zealand) range (a), only quadrats where the tubeworm *Galeolaria caespitosa* were absent in Tasmania and New Zealand (b), and only quadrats in Tasmania, contrasting those with and without *G. caespitosa* (tubeworms; c). The grey areas are 95% confidence intervals derived from bootstrapping of the generalised linear mixed models used to analyse abundance against these predictor variables.

*P. elongatus* was approximately four times more abundant, on average, in its introduced (Tasmania) compared to native (New Zealand) range (Fig. 2a, Table 1). This pattern was unrelated to the structure of its rocky habitat (total boulder surface area, maximum boulder size and mean boulder size), which did not differ between the native and introduced ranges (Fig. 2b; Table 1). In contrast, the cover of tubeworms on the underside of boulders was ~60 times higher in Tasmania than in New Zealand (144.7 ± SE 26.3 and 2.4 ± 1.1 cm^2^ per quadrat pooled across sites, respectively; Fig. 2c; Table 1). Moreover, crab abundances were clearly higher for a given area of boulder habitat in Tasmania compared to New Zealand (Fig. 3a; differences among countries were more pronounced with higher boulder cover: significant country x surface area interaction, χ^2^ = 34.1, P < 0.001). When we removed the influence of tubeworm cover, and only analysed quadrats with boulders lacking tubeworms, the predicted crab abundance across the range of boulder habitat in Tasmania largely overlapped that of New Zealand (95% confidence intervals for cover-abundance relationships, Fig. 3b). Thus, the abundance of *P. elongatus* had the same relationship to available boulder habitat in both its native and introduced ranges. Importantly, when we compared quadrats in Tasmania, the boulders with *G. caespitosa* had a higher abundance of crabs than those lacking tubeworm cover (Fig. 3c, tubeworm effect, χ^2^ = 45.93, *P* < 0.001). Overall, these surveys indicate a strong role for the tubeworm in facilitating the higher abundance of *P. elongatus* in Tasmania compared to New Zealand.

**Table 1:** Contrasts of abundance of crabs, total surface area of boulders, mean boulder size, maximum boulder size and *Galeolaria caespitosa* cover per quadrat among 12 sites in both the introduced range (Tasmania) and native range (New Zealand).

Response variable	Factor	χ^2^	P
Crab abundance	Origin	9.10	0.001
	Site	2203.6	<0.001
Total surface area	Origin	3.13	0.08
	Site	13.01	<0.001
Mean boulder size	Origin	0.19	0.75
	Site	505.35	<0.001
Max boulder size	Origin	0.21	0.65
	Site	44.51	<0.001
*Galeolaria* cover	Origin	10.00	0.006
	Site	20.23	<0.001

For each response variable, analyses were generalised linear mixed models (crab abundance, mean boulder size and G. caespitosa cover) or linear mixed models (total surface area, maximum boulder size) with origin as a fixed factor and site as random factor, the χ2 statistic from likelihood ratio tests, and statistical inference derived from parametric bootstrapping (n = 1000).

**Figure 4:**
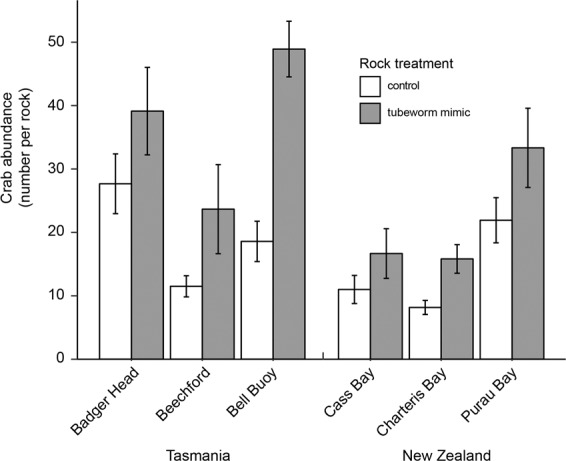
The abundance of *Petrolisthes elongatus* that colonised resin “boulders” (660.5 cm^2^) with an underside surface that mimicked *Galeolaria caespitosa* (tubeworm mimic) and control resin “boulders” with an underside surface that mimicked bare boulder at three sites in each of the introduced (Tasmania) and native (New Zealand) ranges of *P. elongatus*. Data are means ± SE (n = 6–12 boulders per treatment per site).

To explicitly test the effects of the structure provided by *G. caespitosa*, we compared local abundances of colonizing crabs beneath boulder mimics with and without the structure provided by *G. caespitosa*. When placed in the intertidal at three sites in both the native and introduced ranges, the mimics with tubeworm structure always facilitated higher local abundances of *P. elongatus* (by 85% on average across all sites, Fig. 4, Table 2). This facilitation occurred irrespective of the spatial variation in the abundance of *P. elongatus* and any environmental variation among sampling times and sites (adding tubeworm structure increased crab abundance at each of the sites when tested separately, *P* < 0.008 for each of the tests, corrected for multiple comparisons). Even though experiments only ran for 5 days, the mimics with tubeworm structure (Fig. 4) facilitated equivalent numbers of crabs for a similar surface area of boulder (660cm^2^) with tubeworms present in the survey (Fig. 3c). Because the experiments were run at different times in both countries temporal variation in crab abundance between countries may have affected our results. However, we found strong and consistent facilitation of crabs irrespective of region, month, day, local conditions, and adjacent crab densities to provide an extremely robust test of mechanisms underpinning the higher abundances of crabs in Tasmania compared to New Zealand.

**Table 2:** Generalised linear mixed model contrasting the abundance of crabs colonising experimental boulders with either a surface that mimicked *Galeolaria caespitosa* or an unmanipulated surface (boulder treatment) at six sites in Tasmania and New Zealand. Boulder treatment and origin were fixed factors while site was a random factor, the χ^2^ statistic from likelihood ratio tests and statistical inference derived from parametric bootstrapping (n = 1000).

Source	χ^2^	P
Boulder treatment	12.3	0.005
Origin	2.28	0.23
Boulder treatment x Origin	1.00	0.45
Site	6.23	0.01
Site x Boulder treatment	23.73	<0.001

Our short-term experiment determined the immigration of crabs from the surrounding habitat-matrix into our boulders and thus, we may have observed a redistribution of crabs within each site rather than an overall increase in abundance at sites. However, the presence of *G. caespitosa* under boulders in Tasmania also increases the recruitment of juvenile crabs^28^. Thus, it is possible that, at a  population level, the abundance of *P. elongatus* is ultimately affected by the tubeworm because of the immediate increases in local abundances under mimic boulders, and the surveys suggest higher populations in Tasmania are due to presence of the tubeworm. More specifically, we suggest that the higher numbers of crabs under boulders with and without tubeworms in Tasmania, in part, is due to positive demographic feedbacks resulting from high abundances of tubeworms. That is, the tubeworm matrix enhances *P. elongatus* recruitment and supports higher adult abundances leading to greater reproductive output and larger population abundances in its introduced compared to native range.

The impacts of invaders are underpinned by the common assumption that they are more abundant in their introduced range^37^. Whilst this has been demonstrated, most studies, including those investigating positive species interactions in facilitating invader abundance, are conducted in the introduced range only. These studies cannot test whether invaders have an overall greater effect on recipient communities in their native or introduced ranges^37^. Our biogeographic experiment provides crucial *in situ* evidence that native habitat-forming species can facilitate the local abundance of invaders by providing more habitat for the invader than occurs in its native range. An absence of an effect of origin x treatment indicates that higher local abundances in Tasmania are due to the differences in the frequency of facilitation between ranges and not necessarily the intensity of facilitation *per se*. In their introduced range, invaders will generally encounter habitat-forming species that vary in their identity, abundance and/or traits compared to their native range, all of which influence associated community structure. Moreover, in the introduced range, a variety of different native habitat-forming species facilitate the abundance of a diverse range of invaders^25–29^. Thus, differences in facilitation may be a general, but to date understudied mechanism facilitating higher abundances, and potentially the impacts, of invasive species in their introduced compared to native range.

There are a number of direct and indirect mechanisms by which native species can facilitate invaders (see^17,18^). Amelioration of temperature stress by intertidal ecosystem engineers is a critical process facilitating their associated biota^12,42^ and is likely an important mechanism promoting the local abundance of *P. elongatus* when associated with *G. caespitosa*. The presence of *G. caespitosa* on the underside of boulders reduces temperature by up to 6 °C compared to boulders without *G caespitosa*^33^. However, the tubeworm matrix may also modify other abiotic and biotic processes such as reducing predation pressure^43^, trapping larvae (and thus enhancing propagule pressure) or food particles or providing a more rugose structure for *P. elongatus* to cling onto^35^.

Higher abundances of invasive species in their introduced compared to native ranges are often related to changes in negative species interactions such as a reduction in the effects of natural enemies or increased competitive ability^3^. This is predicted to result in shifts in body size and higher reproductive output, and therefore higher fitness, in the introduced compared to native range^44,45^. Indeed, another invasive porcelain crab, *P. armatus*, has a smaller size at first reproduction and increased reproductive output in invasive populations on the southeastern coast of the USA compared to native populations in Brazil^46,47^, although it is unlikely that *P. armatus* on the southeastern coast of the USA came from Brazil^48^. We do not know whether invasive *P. elongatus* have higher fitness, however, male *P. elongatus* are larger in Tasmania compared to New Zealand although females are not^34^. Thus, although we observed strong positive effects of the mimics with tubeworm structure present in both countries, we cannot discount changes in other factors in (e.g. negative biotic interactions) in contributing to the higher abundance of *P. elongatus*  in Tasmania compared to New Zealand.

In this study, we used standardised structural mimics to present the first unconfounded *in situ* experiment (*in situ* tests for shifts in biotic interactions typically contain different biotic communities in the different ranges or utlise common garden approaches) addressing one of the most challenging and enduring questions in invasion ecology: why are many invaders more abundant in their introduced ranges? Invasion research has focused overwhelmingly on reductions in enemy effects to explain higher invader abundances in the introduced compared to the native range. By comparison, our study provides novel evidence that increases in habitat provided by native species may also explain these patterns. There are many positive interactions that might benefit invaders, including those provided by habitat-forming species (e.g. via ameliorating abiotic stress). We argue that these positive effects should be incorporated more explicitly into invasion ecology theory to broaden our understanding of the processes driving the abundance and distribution of invasive species across their native and introduced ranges.

## Methods

### Survey of crab abundances and habitat-characteristics in Tasmania and New Zealand

The abundance of *P. elongatus* and habitat-characteristics were determined at six sites in both New Zealand and Tasmania (Fig. 1). All sites were relatively similar and homogeneous being characterized by low vertical relief with a rocky substrate and boulders with numerous interstitial spaces^34^. The introduced range of *P. elongatus* in Tasmania is primarily on wave-protected shores on the north coast and sheltered embayments on the east coast^32,34,49^. To minimize any temporal effects of sampling, all sites were surveyed between April and June 2015.

To contrast the abundance of *P. elongatus* between Tasmania and New Zealand, we sampled the intertidal (from low to mid zones) regions at all sites. Within sites, 18 replicate quadrats (0.5 ×0.5 m) were haphazardly positioned (avoiding a few scattered very large boulders that would be impossible to turn around). The abundance of crabs under boulders was determined by removing all the boulders within each quadrat, catching crabs by hand and counting them^34^. Newly settled megalopae were not included as they were too small to accurately count. The number of crabs per quadrat was contrasted between the native and introduced range using a generalised linear mixed model with origin (native vs. invasive) as a fixed factor, site as a random factor and a Poisson error distribution. Origin was considered a fixed factor as *P. elongatus* is only invasive in Australia and only native in New Zealand. The analyses were run in the R package lme4^50^ with statistical inference of effects from parametric bootstrapping (n = 1000) of the likelihood ratio statistic contrasting full and reduced models.

To quantify differences in habitat characteristics between the native and introduced range of *P. elongatus*, we measured the total underside boulder surface area, mean underside boulder size, maximum underside boulder size and total underside tubeworm cover from the same quadrats sampled for *P. elongatus*. For each quadrat, the boulders that were removed were placed upside down in a tray and a photograph was then taken of the underside of all boulders. Measurements were taken from photos using the image analysis software, ImageJ (https://imagej.nih.gov/ij/index.html). Total underside boulder surface area per quadrat and maximum boulder size were contrasted between the native and introduced range with linear mixed models with origin as a fixed factor and site as a random factor. Mean underside boulder size and total underside tubeworm cover per quadrat were contrasted between the native and introduced range with generalised linear mixed models with origin as a fixed factor, site as a random factor and a negative binomial error distribution.

To explore the influence of boulder and tubeworm cover on crab abundance, we ran three further models to determine: 1) the relationship between total underside boulder surface area and crab abundance in both Tasmania and New Zealand, 2) the relationship between total underside boulder surface area and crab abundance in both countries when *G. caespitosa* was absent, and 3) the relationship between underside boulder cover and crab abundance in Tasmania only, comparing boulders with and without *G. caespitosa*. Each of these was a generalised linear mixed model with site as a random factor, total underside boulder surface area as a fixed factor, origin (models 1 and 2) or *G. caespitosa* present/absent (model 3) as a fixed factor, and a Poisson error distribution.

### Effect of tubeworm presence on *P. elongatus* abundance in New Zealand and Tasmania

We conducted a biogeographic experiment in the introduced and native ranges of *P. elongatus* to determine the effects of the tubeworm on crab abundance. More specifically, because habitat-formers influence associated communities largely by their structure^15,36^, we here only tested for structural tubeworm effects. To do this, we made realistic structural mimics of the undersides of boulders with and without *G. caespitosa* present (Supplementary Fig. S1).

To construct the mimics, we selected a boulder from Bell Buoy Beach in Tasmania that had>95% cover of tubeworm matrix on its bottom surface, was clean of other sessile organisms and had equal surface area on the top and bottom (660.52 cm^2^) of the boulder. The boulder was left to dry for 5 days. Separate mimics of the bottom and the top of the boulder were made to create mimics with and without the tubeworm matrix, respectively. Detailed methods of the construction of the mimics can be found in the Supplementary Text. Briefly, mimics were constructed by pouring polyurethane into casts of the top and bottom of the boulder made from a silicone mould. The sides of the mimics were smooth and 4 cm high with a hollow well on the side opposite the mimic side (in which we could add boulders and thereby fix the mimics in space, see also Supplementary Fig. S2). We created 12 replicate mimics each of the top and bottom of the boulders although the number of mimics for each treatment differed among sites because some were overturned and some were lost during the experiments (see below).

Identical experiments using the mimics were conducted at three sites in both Tasmania and New Zealand (Fig. 1). In Tasmania, the experiments were conducted at Bell Buoy Beach (from November 15–21, 2016; N = 12 and 11 replicate smooth and complex mimics recovered, respectively), Badger Head (from December 4–10, 2017; N = 9 and 8 replicate smooth and complex mimics recovered, respectively) and Beechford (from December 10–16, 2017; N = 6 and 6 replicate smooth and complex mimics recovered, respectively) (Fig. 1). In New Zealand, the experiments were conducted at Purau Bay (from May 10–17, 2017; N = 12 and 12 replicate smooth and complex mimics recovered, respectively), Cass Bay (from June 24–30, 2017; N = 9 and 9 replicate smooth and complex mimics recovered, respectively) and Charteris Bay (from December 14–21, 2016; N = 12 and 11 replicate smooth and complex mimics recovered, respectively). Although sites in New Zealand were closer together, they were all at least 10 kms apart. At each site, mimics were haphazardly placed into the intertidal boulder fields in areas that contained *P. elongatus* but avoiding depressions where water accumulated to standardise the underlying substrate as much as possible. The two treatments were interspersed to avoid spatially confounding effects and once a mimic was positioned, a large boulder (>10 kg) was placed in the well on the top of the mimic to hold it in place. Mimics were left in place for five days which is sufficient time for immigration of *P. elongatus* under boulders. We did not observe *P. elongatus* colonising the tops of the mimics under the boulder weights. The abundance of *P. elongatus* beneath each mimic was determined using high speed video. Briefly, a video that captured both the bottom surface of the boulder and the substrate where the boulder had been was taken as the boulder was turned over. Videos were taken with a Nikon Coolpix Aw110 camera set at 30 frames per second in 720p format and there is a strong positive relationship between video and hand capture techniques for *P. elongatus* abundance (R² = 0.960, P < 0.001^35^). All mimics were cleaned thoroughly after each experiment, and there was no evidence of mimics eroding during the experiments.

The number of crabs per boulder was contrasted among treatments with a generalised linear mixed model with origin and boulder treatment (with and without the tubeworm matrix) as fixed factors, site as a random factor and a Poisson error distribution. We found a significant site x boulder treatment interaction (*P* < 0.001; Table 2) and therefore used generalised linear models to contrast treatments separately within each site, using a Bonferroni correction for multiple comparisons.

## Supplementary information

Supplementary Information.
