# Supplementary material for: Habitat provided by native species facilitates higher abundances of an invader in its introduced compared to native range
Source: Sci Rep. 2020 Apr 14;10:6385. doi: 10.1038/s41598-020-63429-2 (PMC7156459; doi:10.1038/s41598-020-63429-2)
Supplement: Supplementary file 1 — Supplementary Information. [file 41598_2020_63429_MOESM1_ESM.docx]

**Habitat provided by native species facilitates higher abundances of an invader in its introduced compared to native range**

Paul E. Gribben, Alistair G. B. Poore, Mads S. Thomsen, Phoebe Quesey, Emma Weschke and Jeffrey T. Wright

| A | 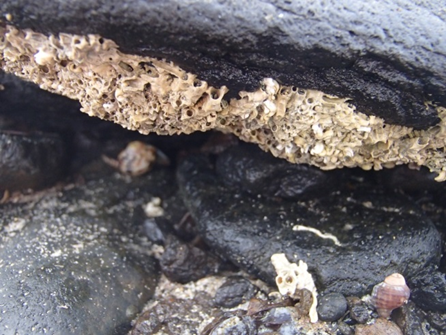 | B | **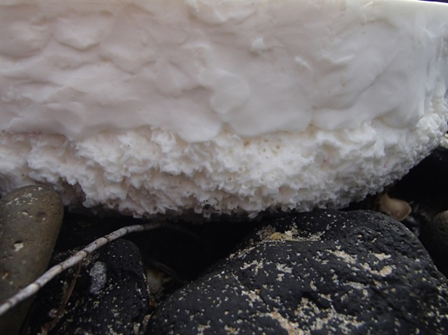** |
| --- | --- | --- | --- |

Supplementary Figure S1. Photos of structure provided by *Galeolaria caespitosa* underneath boulders (A) and the equivalent structure provided by the polyurethane mimics (B).

**Supplementary Text**

**Production of boulder mimics**

A boulder was selected from Bell Buoy Beach (41° 02' 25"S, 146° 49' 58"E), Tasmania with >95% cover of *Galeolaria caespitosa* tube matrix on the underside and bare on the topside. The boulder was removed and allowed to dry for 5 days for sterilisation purposes. The boulder was transported from Hobart, Tasmania to UNSW, Sydney on January 27^th^ 2016.

The same process was used to make moulds of the sides of the boulders with and without the *G. caespitosa* matrix present so we only describe the process for creating the *G. caespitosa* present mimics (Fig. S2A-E). First, the boulder was placed on clay which sat on a flat metal surface with the *G. caespitosa* cover facing up. To isolate the *G. caespitosa* covered side of the boulder, clay was sculpted vertically down the sides of the boulder covering and concealing the bare underside (Fig. S2A). A wall of clay was built around the boulder ~15 mm distance away from the boulder, and was made at least 20 mm higher than the highest point of the boulder (Fig. S2B). The external clay wall was then wrapped in plastic wrap and covered the external wall to ensure that no leakage of the liquid silicone once it was poured into the mould (Fig. S2B).

Sealant was sprayed onto the boulder and clay to allow careful removal of the silicone mould from the matrix, thus preventing silicone from getting stuck inside the intricate tubing. The silicone was poured into the space enclosed by boulder and clay with care to remove air spaces between the silicone and the boulder/tubes (Fig. S2B). We used PinkySil® silicone moulding rubber due to its elasticity and ability to achieve a high level of detail and complexity. The silicone was left to cure for two days before pulling it off the boulder (Fig. S2C). We then designed a silicone plug to place within the silicone mould to create a cast with a hollow centre (Fig. S2D).

Following construction of the moulds, casts of moulds were produced using TC-808 78 Shore D Urethane Casting System White. These plastics form casts that have the hardness of the CaCO_3_ tube matrix. This material resists damage from natural physical disturbance such as wave action, and will not dissolve in saltwater. The colour was white to mimic the calcareous tube matrix. The polyurethane was poured into the mould in parts after a layer of sealant was sprayed onto the moulds to minimise tearing of the delicate tubes (Fig. S2D). Initially, a 2 cm base was poured into the mould to create a mimic surface and allowed to cure for 30 mins. Then a silicone plug was placed on the hardened polyurethane surface and the space between the plug and the sides of the silicone mimic filled with polyurethane to form the walls of the polyurethane mimic (Fig. S2D). Demoulding was carried out after ~30 minutes of cure time for the polyurethane (Figs. S2E). In total we made 12 casts from each of the two moulds.

| A | 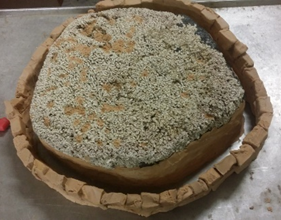 | B | 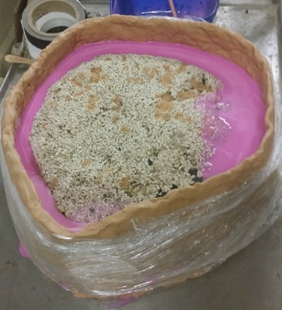 |
| --- | --- | --- | --- |
| C | 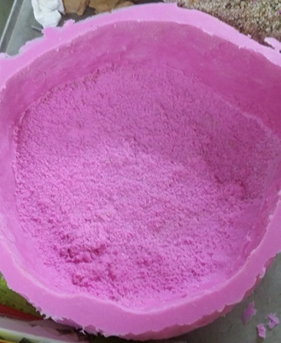 | D | 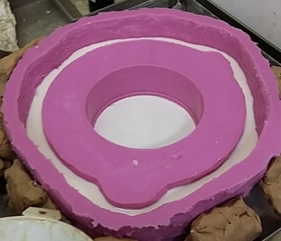 |
| E | 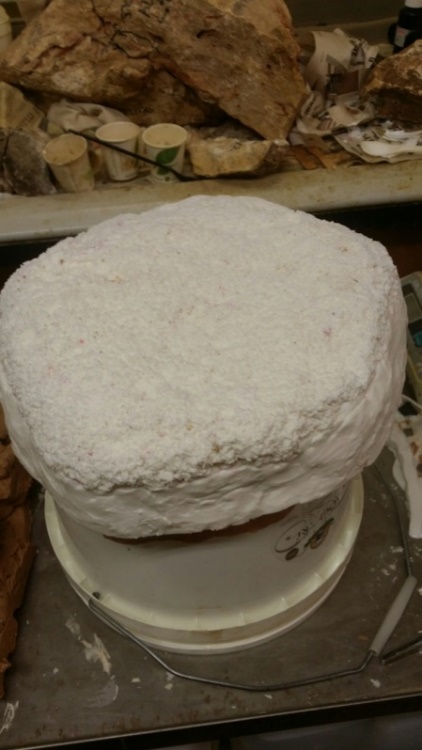 |  |  |

Supplementary Figure S2. Photos of the construction of mimics of *Galeolaria caespitosa* on boulders showing moulding of clay to isolate *G. caespitosa* side of the boulder pouring of silicone to create mimic of *G. caespitosa* surface (B), finished silicone mimic of *G. caespitosa* (C), polyurethane mimic with the silicone plug still in place and (D) and a bottom view of the finished *G. caespitosa* mimic (E).
